# Characteristics and Surgical Results of Patients with Hypertrophic Obstructive Cardiomyopathy without Intrinsic Mitral Valve Diseases Undergoing Mitral Subvalvular Procedures during Myectomy

**DOI:** 10.1155/2020/8875405

**Published:** 2020-12-09

**Authors:** Qiang Ji, YuLin Wang, Ye Yang, LiMin Xia, WenJun Ding, Kai Song, ChunSheng Wang

**Affiliations:** ^1^Department of Cardiovascular Surgery of Zhongshan Hospital Fudan University, 180 Fenglin Road, Shanghai 200032, China; ^2^Department of Cardiovascular Surgery of Xiamen Branch of Zhongshan Hospital Fudan University, 668 Jinhu Road, Huli District, Xiamen 510530, China; ^3^Shanghai Institute of Cardiovascular Diseases, 1609 Xietu Road, Shanghai 200032, China

## Abstract

**Background:**

Mitral subvalvular procedures have acquired a major role during hypertrophic obstructive cardiomyopathy (HOCM) surgery. However, few studies have focused on characterizing the clinical feature of HOCM patients without intrinsic mitral valve (MV) diseases undergoing mitral subvalvular procedures in addition to myectomy. Additionally, scant data about the results of mitral subvalvular procedures during HOCM surgery are available. This single-center study aims to characterize the clinical feature and surgical results of HOCM patients without intrinsic MV diseases undergoing mitral subvalvular procedures in addition to myectomy in comparison with those receiving myectomy alone.

**Methods:**

Among 181 eligible patients, 50 (27.6%) patients undergoing myectomy plus mitral subvalvular procedures were entered into the combined group, and the remaining 131 patients receiving myectomy alone were included in the alone group. Baseline and surgical characteristics were investigated, and surgical results were compared.

**Results:**

Comparatively, the combined group was younger (52.9 ± 11.2 years vs. 56.8 ± 11.8 years, *p*=0.045) and had a better New York Heart Association (NYHA) class (*p*=0.034) and less septal hypertrophy (16.4 ± 2.3 mm vs. 18.5 ± 3.2 mm, *p* < 0.001). Septal thickness was independently associated with combined procedures in multivariable logistic regression analysis (OR = 0.887, 95% CI 0.612–0.917). No surgical death or iatrogenic septal perforation occurred in the combined group. Two (6.5%) patients in the combined group developed complete atrioventricular block and required permanent pacemaker implantation. During a median follow-up of 10 months, no deaths or reoperations were observed with the symptom of relief and NYHA class I or II in either group. Patients in the combined group as compared to the alone group had lower outflow tract gradients and a lower incidence of residual systolic anterior motion (SAM) syndrome.

**Conclusions:**

For HOCM patients without intrinsic MV diseases who are scheduled for surgery, a less pronounced septal hypertrophy may be closely associated with myectomy with concomitant mitral subvalvular procedures instead of myectomy alone. Mitral subvalvular procedures during myectomy are safe and allow the reduction of outflow tract gradients and freedom from SAM more effectively in comparison with myectomy alone.

## 1. Introduction

Hypertrophic cardiomyopathy (HCM) is a genetic myocardial disease with the characterization of asymmetrical left ventricular hypertrophy [1]. Up to 70% of HCM patients develop hypertrophic obstructive cardiomyopathy (HOCM) characterized by severe left ventricular outflow tract (LVOT) obstruction at rest or after provocation [2], which is frequently associated with an unfavorable prognosis [3]. Septal myectomy has been a standard treatment option for HOCM patients with drug refractory symptoms who are scheduled for septal reduction therapy [4–7].

According to Mayo's experience [8], myectomy alone may be sufficient in the relief of LVOT obstruction and in the elimination of mitral regurgitation (MR) in the overwhelming majority of HOCM patients, and concomitant mitral valve (MV) surgery has been required only in 2.1% of HOCM patients without intrinsic MV diseases. In contrast, increasing studies [9, 10] have reported that mitral subvalvular anomalies, including fibrotic anterior leaflet attachment and abnormalities of the papillary muscles (PMs) and secondary chordae [11], may play an important role in the etiology of HOCM, and myectomy alone may receive incomplete or only temporary relief of LVOT obstruction in a subgroup of HCM patients without intrinsic MV diseases. Mitral subvalvular apparatus has been considered playing a major role in patients with a minor degree of septal hypertrophy without intrinsic MV diseases [11]. As its importance is recognized, mitral subvalvular procedures have acquired a major role during HOCM surgery [12].

The majority of previous studies have paid attention to assess the role of mitral subvalvular apparatus in the etiology of HOCM and to evaluate the results of MV procedures during HOCM surgery. However, few studies have focused on characterizing the clinical feature of HOCM patients without intrinsic MV diseases undergoing mitral subvalvular procedures in addition to myectomy. Additionally, scant data about the results of mitral subvalvular procedures during HOCM surgery are available.

This single-center study aims to characterize the clinical feature and surgical results of HOCM patients without intrinsic MV diseases undergoing mitral subvalvular procedures at the time of myectomy compared with those receiving myectomy alone, to provide reference for surgical decision-making of symptomatic HOCM.

## 2. Methods

### 2.1. Patients

Between January 2016 and July 2019, documented HOCM patients aged ≥18 years with drug refractory symptoms who met the indications for operation according to the European Society of Cardiology guidelines [13] and underwent septal myectomy with or without concomitant mitral subvalvular procedures in this center were reviewed. The exclusion criteria were as follows: (1) intrinsic MV diseases (rheumatic, degenerative, infective, and mitral annulus calcification); (2) previous alcohol septal ablation therapy; (3) concomitant other valvular heart disease requiring surgery; (4) concomitant coronary artery disease requiring bypass grafting; (5) concomitant maze IV procedure; and (6) implanted cardioverter defibrillator. This study did not include patients with septal hypertrophy secondary to hypertensive heart disease or severe aortic valvular stenosis.

### 2.2. Preoperative Echocardiography Evaluation

**Figure 1:**
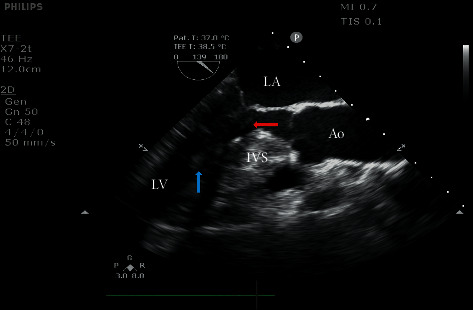
Mitral subvalvular abnormalities.

Transthoracic echocardiography (TTE) examination was performed within 3 days before surgery to define (1) the location and magnitude of any left ventricular pressure gradient, both at rest and with provocation; (2) the distribution and severity of myocardial hypertrophy; (3) MV anatomy and function; and (4) the presence of mitral subvalvular anomalies. Resting LVOT velocity was measured by continuous-wave Doppler of the outflow tract from an apical window, and the resting LVOT pressure gradient was estimated by using the modified *Bernoulli* equation (i.e., gradient = 4v^2^, where *v* = peak LVOT velocity). In symptomatic patients with resting LVOT gradients <30 mmHg, maneuvers such as the Valsalva maneuver and stand-to-squat were frequently used prior to repeat TTE examination. Mitral subvalvular anomalies included abnormal chordae tendineae attached to the ventricular septum or free wall (false cords), PM abnormalities (hypertrophy, fusion, direct insertion into the anterior MV leaflet, aberrances (e.g., bifurcation and fibrosis), and accessory PM), and fibrotic and retracted secondary chordae inserted on the anterior mitral leaflet body, as shown in Figure 1.

### 2.3. Study Protocol

This study protocol was approved by the Ethics Committee of Zhongshan Hospital Fudan University (no. Y2020-029) and was consistent with the Declaration of Helsinki. All included patients signed an informed consent approved by the ethics committee. Data collection was performed by trained staff (two people). The trained staff, however, were not informed of the purpose of this study.

All included patients were entered into a combined group (patients undergoing mitral subvalvular procedures at the time of myectomy) or an alone group (patients receiving myectomy alone). Baseline and surgical characteristics and perioperative results were obtained from our institutional database and were reviewed using a standard data collection form. Perioperative results included unplanned ventricular septal defect repair, unplanned aortic valve operation, unplanned MV operation, unplanned repair of left ventricular rupture, surgical death, major postoperative morbidity (atrial fibrillation, ventricular fibrillation, prolonged ventilation (>48 hours), deep sternal infection, stroke, renal failure, and reoperation), and permanent pacemaker insertion. Surgical death was defined as any death within postoperative 30 days or within the same hospitalization.

Patients were regularly followed up at 3 and 6 months following surgery and in 6-month intervals thereafter. Follow-up data, including survival, reoperation, New York Heart Association (NYHA) functional class, maximum LVOT gradient, residual obstruction, systolic anterior motion (SAM), severity of MR, and residual MR, were obtained through clinic visits, WeChat, or telephone interviews. Residual obstruction was defined as a maximum LVOT gradient of >30 mmHg measured by TTE, and residual MR was defined as moderate or high MR during follow-up.

### 2.4. Surgical Procedures

Intraoperative transesophageal echocardiography (TEE) was performed after the induction of anesthesia for the estimation of the MV lesion and mitral subvalvular apparatus and modeling of an adequate length and depth of resection into the LVOT. The patients were gently placed on the reverse Trendelenburg in the left lateral decubitus position. After a median incision with sternotomy, cardiopulmonary bypass was performed using ascending aortic cannulation and right atrium cannulation with a left ventricular vent placed via the right superior pulmonary vein. Through a low oblique aortotomy approximately 7–10 mm above the right coronary ostium, the aortic valve leaflets were pulled up to gain access to the outflow tract. Scalpel resection was usually started at the nadir of the right cusp, 5 mm below the aortic valve and extending leftwards to the left trigone. The area of septal excision was lengthened beyond PM root and toward the apex of the heart. The depth of resection was up to 50% of the basal thickness of the septum.

In addition to septal myectomy, mitral subvalvular anomalies were corrected if they existed. The attachments (fibrous or muscular) between the subvalvular apparatus and septum or free wall were divided, but all attachments to the leading edge of the anterior leaflet were preserved to avoid iatrogenic MV incompetence. Transaortic resection of fibrotic, thickened, and agglutinated secondary chordae tendineae was performed from the tip of the PM to the ventricular surface of the anterior MV leaflet. In addition, the PMs were carefully inspected to detect any hypertrophy, fusion, displacement, anomalies, or aberrances (e.g., bifurcation and fibrosis). Hypertrophied and thick PMs were split to increase mobility. Accessory PMs were resected.

TEE was used after weaning off bypass to assess the provoked gradient, SAM, and residual MR following myectomy. Provocation was frequently conducted by isoproterenol infusion via a deep venous catheter to achieve a heart rate greater than 120 beats per minute or induce premature ventricular contraction via mechanical stimulation of the right ventricle during the TEE examination. A repeat procedure was immediately performed if there was residual obstruction and/or residual moderate or more MR or if a ventricular septal perforation or a left ventricular free wall rupture was observed.

### 2.5. Statistical Analysis

Statistical analysis was performed with the SPSS statistical package version 22.0 (SPSS Inc., Chicago, IL, USA). Categorical data were expressed as frequency distributions and single percentages and were compared between groups using Fisher's exact test if the expected frequency was <5 or the chi-square test. Normally distributed continuous variables were expressed as the mean ± standard deviation and were compared between groups using an independent-samples *t* test; non-normally distributed continuous variables were expressed as median and interquartile range (IQR) and were compared between groups with the *Wilcoxon* rank sum test. Baseline characteristics (including demographics, concomitant diseases, preoperative cardiac status, and echocardiographic data) with *p* < 0.2 obtained through the univariate analysis were then entered into forward stepwise multivariate logistic regression analysis (the combined group or the alone group as independent variables and baseline variables as dependent variables) to test the independent association between grouping (concomitant mitral subvalvular procedures vs. myectomy alone) and baseline characteristics. A two-sided *p*-value less than 0.05 was considered statistically significant.

## 3. Results

### 3.1. Study Population

A total of 234 adult patients who met the inclusion criteria were reviewed. Two patients with septal hypertrophy secondary to severe aortic valvular stenosis were not included, and another 51 patients were excluded due to concomitant intrinsic MV diseases requiring surgery (30), previous alcohol septal ablation therapy (12), concomitant maze IV procedure (5), and concomitant coronary artery bypass grafting (4), leaving 181 eligible patients (97 males, with a mean age of 55.7 ± 11.8 years) for data analysis. Although 39.2% of the population had a history of hypertension, hypertension was not deemed severe enough to be the primary cause of ventricular hypertrophy. All patients manifested severe limiting symptoms refractory to optimal medical therapy with nonvasodilating *β*-blockers and/or calcium channel blockers, with the NYHA functional classes III and IV in 86.7% of the population.

In this series, 50 (27.6%) patients undergoing mitral subvalvular procedures in addition to myectomy were entered into the combined group, and the remaining 131 patients receiving myectomy alone were included in the alone group.

### 3.2. Baseline and Surgical Characteristics

The baseline characteristics are listed in Table 1. Comparatively, the combined group was younger (52.9 ± 11.2 years vs. 56.8 ± 11.8 years, *p*=0.045) and more likely to be having family history of sudden death (8.0% vs. 1.5%, *p*=0.049) and had a better NYHA functional class (*p*=0.034) but shared a similar proportion of male patients and comparable concomitant other diseases. The TTE evaluation within three days before myectomy showed that patients in the combined group had a maximum gradient of 96.5 ± 17.8 mmHg, which was similar to the alone group (*p*=0.193). Compared to those in the alone group, patients in the combined group had a smaller degree of septal hypertrophy (septal thickness, 16.4 ± 2.3 mm and 18.5 ± 3.2 mm, *p* < 0.001). Importantly, all patients in the combined group had mitral subvalvular anomalies, including false cords (13 patients), fibrotic and retracted secondary chordae (34 patients), and PMs abnormalities (17 patients). Accordingly, patients in the combined group underwent mitral subvalvular procedures, including false cords cutting (*n* = 13, 26.0%), secondary chordae cutting (*n* = 34, 68.0%), and PM release and/or resection (*n* = 17, 34.0%) in addition to myectomy (*n* = 50, 100%). In the combined group, 68.0% of patients underwent fibrotic and retracted secondary chordae cutting; typically, 1 to 3 secondary chordae were resected depending on individual anatomy. 34.0% of patients from the combined group underwent longitudinal PM resection in addition to the mobilization technique. The combined group as compared to the alone group showed a trend toward longer aortic cross-clamping time (38.1 ± 7.3 min vs. 35.8 ± 7.5 min, *p*=0.065).

Baseline characteristics with *p* < 0.2 obtained through the univariate analysis were age, males, recent smoking, family history of sudden death, NYHA class III and IV, the maximum gradients, and septal thickness. These baseline variables were then entered into forward stepwise multivariate logistic regression analysis (surgical type as independent variables and baseline variables as dependent variables) to test the independent association between surgical type (concomitant mitral subvalvular procedures vs. myectomy alone) and baseline characteristics. As shown in Table 2, only septal thickness was a predictor of mitral subvalvular procedures in addition to myectomy in stepwise multivariable logistic regression analysis (OR = 0.887, 95%CI 0.612–0.917, *p*=0.005).

### 3.3. Intraoperative Outcomes

As shown in Table 3, immediate repeat surgery was recorded for 3 (6.0%) patients from the combined group and 7 (5.3%) from the alone group (*p*=0.391). In the combined group, one patient developed residual obstruction with a provoked gradient of more than 40 mmHg and moderate-severe MR due to inadequate initial septal myectomy, and then, underwent a “more” extended myectomy according to the TEE findings; another underwent repair of iatrogenic left ventricular free wall rupture; and the remaining one received aortic right valve repair due to iatrogenic perforation. In the alone group, 4 patients underwent immediate repeat surgery (including 2 patients with “more” extended myectomy, one with MV repair using the “edge-to-edge” technique, and another one with anterior leaflet enlargement using patch) due to inadequate initial myectomy, and the remaining 3 patients underwent immediate repeat surgery due to iatrogenic left ventricular wall rupture (2 patients) and iatrogenic septal perforation (one patient).

The TEE examination immediately after weaning off bypass showed that the maximum LVOT gradients following myectomy fell to 8.1 ± 5.2 mmHg in the combined group and to 11.9 ± 7.0 mmHg in the alone group (*p* < 0.001 for comparison between groups), both of which were significantly lower than the preoperative values (both *p* < 0.001). The septal thickness following myectomy was significantly smaller than that preoperatively in both groups (both *p* < 0.001), with no significant difference between groups (13.7 ± 1.7 mm vs. 14.3 ± 2.6 mm, *p*=0.132). Patients in both groups achieved significant improvements in the severity of MR following myectomy (both *p* < 0.001). SAM following myectomy was identified in 17 patients, with a lower incidence in the combined group (0 vs. 13.0%, *p*=0.007).

### 3.4. In-Hospital Outcomes

No surgical death occurred in the combined group; nevertheless, one (0.8%) patient from the alone group died of cerebral hernia on the fourth day postoperatively, which may be associated with acute cerebral infarction. No significant difference was recorded between groups regarding the surgical death (*p*=0.536).

A total of 6 patients (2 from the combined group vs. 4 from the alone group, *p*=0.669) developed a complete atrioventricular node block and required permanent pacemaker implantation. Note that among the 6 patients, 3 patients (one from the combined group and 2 from the alone group) had right bundle branch block prior to surgery. In addition, 5 patients (4.0% vs. 1.7%, *p*=0.617) developed new-onset atrial fibrillation, but all the 5 patients returned to sinus rhythm following electrical cardioversion. The other postoperative complications are listed in Table 3. A total of 180 patients were discharged, and the median length of postoperative hospital stay was 6 days for the combined group and 6 days for the alone group (*p*=0.412).

### 3.5. Follow-Up Outcomes

**Figure 2:**
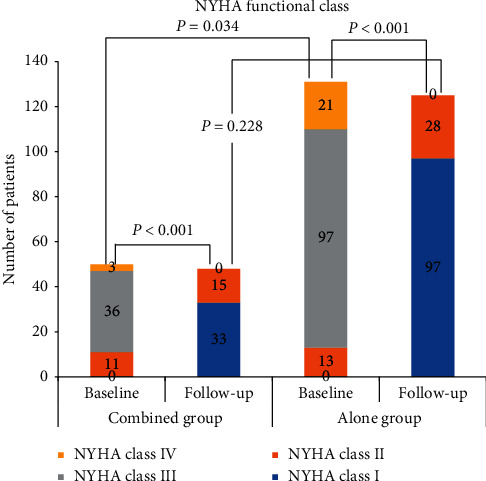
NYHA functional status before myectomy and at the latest follow-up.

**Figure 3:**
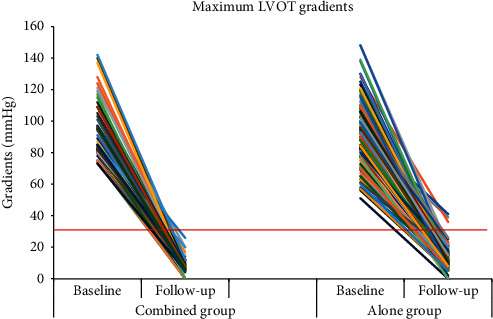
Maximum LVOT gradients before myectomy and at the latest follow-up. The red line represents a gradient level of 30 mmHg. LVOT, left ventricular outflow tract.

A total of 173 patients (48 from the combined group and 125 from the alone group) received a follow-up visit with a median duration of 10 (IQR, 6–15) months. No significant difference between the two groups was observed regarding follow-up time (median and IQR, 9 (6–15) months vs. 10 (6–15) months, *p*=0.312). During the follow-up periods, the clinical manifestations disappeared, and no deaths or reoperations were observed. NYHA functional class significantly decreased from the preoperative value in both groups, with no patients being class III or IV and no significant difference between groups (Figure 2). As shown in Figure 3, the provoked gradients at the latest follow-up were significantly lower than the preoperative values in both groups; also, residual obstruction was not observed in the combined group or was recorded in 3 (2.4%) patients from the alone group (*p*=0.279). SAM was identified in 11 patients via the TTE examination at the latest follow-up, of whom 3 were diagnosed with moderate MR and the remaining 8 were diagnosed with mild or less MR. There were no cases of severe MR at follow-up. Comparatively, patients in the combined group had a lower incidence of the SAM syndrome (0 vs. 8.8%, *p*=0.034) despite no significant difference in the incidence of moderate residual MR (0 vs. 2.4%, *p*=0.279). None had MV flail or prolapse at the most recent evaluation. No instances of moderate or more aortic regurgitation were observed. Note that one patient from the nonlatent group who developed iatrogenic septal perforation intraoperatively was found to have one 2-mm asymptomatic ventricular septal defect during follow-up. The patient was categorized as NYHA class I, and dynamic evaluation was continued.

## 4. Discussion

A minority of HCM patients with significant LVOT gradients has relatively mild septal hypertrophy, which can make it technically difficult to achieve an adequate reduction in muscle thickness via myectomy alone [7]. Previously, a subset of such patients underwent MV replacement as an alternative to myectomy [7, 14, 15]. In addition to extension or plication of the anterior MV leaflet [16, 17], mitral subvalvular procedures, which aimed at freeing the posterior motion of the anterior mitral leaflet away from the septum and moving back the coaptation plane of the mitral valve, in order to abolish the SAM syndrome and LVOT obstruction [8, 9], have been conducted in the subgroup of HOCM patients with relatively mild septal hypertrophy without intrinsic MV diseases to avoid MV replacement and the complications associated with prosthetic valves. Mitral subvalvular procedures included false cords cutting, fibrotic and retracted secondary chordae cutting, and PM release and/or resection. Abnormal chordae tendineae that attach to the ventricular septum or free wall (“false cords”) may contribute to LVOT obstruction and should be cut. Thickened and retracted secondary chordae that insert beyond the free margin and rough zone of the anterior mitral leaflet may cause abnormal tethering of the anterior MV leaflet and favor the displacement of slack portions of the leaflet (and attached primary chordae) into the LVOT and the ejection flow and predisposing to SAM [8, 9]. The distal extreme of the secondary chordae was cut from its connection to the papillary muscle, and the proximal extreme was cut from the ventricular surface of the anterior MV leaflet. Resected chordae were particularly thickened, often agglutinated, and usually connected to a fibrotic area of the corresponding papillary muscle. In addition, PM anomalies, including direct PM insertion into the anterior mitral leaflet, extensive fusion of PM to the ventricular septum or left ventricular free wall, and accessory PMs, all of which can tether the MV toward the septum and contribute to LVOT obstruction, have been frequently observed in HOCM patients [11, 14, 18]. In most cases, all anomalous muscle bundles were removed, and aberrant adhesions were detached. However, thin muscle bundles connecting anterior commissure and adjacent septum should be reserved if they exist [19].

An important finding of this study was that HOCM patients without intrinsic MV diseases undergoing myectomy with concomitant mitral subvalvular procedures in comparison with those undergoing myectomy alone had less septal thickness. This study reviewed 181 HOCM patients without intrinsic MV diseases, including 50 undergoing mitral subvalvular procedures at the time of myectomy (the combined group) and 131 undergoing myectomy alone (the alone group), and found that patients in the combined group as compared to the alone group had a smaller degree of septal hypertrophy in a univariate analysis. Furthermore, septal thickness was a predictor of combined procedures via multivariate logistic regression analysis. This result suggested HOCM patients with combined procedures may have less septal thickness. Alternately, for HOCM patients without intrinsic MV diseases, the less pronounced the septal hypertrophy, the more likely the combined procedures should be undergone instead of myectomy alone. Asymmetric marked hypertrophy of the interventricular septum has been recognized as a key cause of LVOT obstruction [5, 7]. However, a minority of HCM patients without intrinsic MV diseases had relatively mild septal hypertrophy but manifested significant LVOT gradients [7]. In such patients, septal hypertrophy may not be the sole cause of LVOT obstruction [12, 14]. Previous studies have reported that mitral subvalvular anomalies can tether the mitral leaflets toward the septum and contribute to LVOT obstruction [12, 14]. Less septal hypertrophy has been reported to be more associated mitral subvalvular anomalies [12, 20]. So, HOCM patients with less septal hypertrophy without intrinsic MV diseases may have abnormalities of the mitral subvalvular apparatus, which contributed importantly to LVOT obstruction [14, 15] and may require mitral subvalvular procedures during myectomy. These evidences were in line with the result of this study. Our results suggested that for HOCM patients without intrinsic MV diseases, a less pronounced septal hypertrophy may be closely associated with myectomy with concomitant mitral subvalvular procedures instead of myectomy alone. Therefore, for HOCM patients with less septal hypertrophy without intrinsic MV diseases, increased awareness of the importance of mitral subvalvular anomalies and an improved ability to visualize subvalvular structures by means of optical magnification and fiber optic lighting may lead to increased identification and successful surgical treatment. This study stressed once again the importance of mitral subvalvular procedures for HOCM patients, especially for those with a minor degree of septal hypertrophy without intrinsic MV diseases.

Another important finding of this study was that myectomy with concomitant mitral subvalvular procedures achieved excellent results and allowed the reduction of LVOT gradient and freedom from SAM syndrome more effectively in comparison with myectomy alone. In each of the patient with mitral subvalvular procedures in addition to myectomy, functional limitation decreased to class I or II with the symptom relief, and LVOT obstruction was completely abolished, and SAM syndrome was completely abolished with no moderate or more residual MR. None of the patients with combined procedures developed death or reoperation during follow-up. An improvement in NYHA functional class and symptom coincided with complete abolishment of LVOT obstruction and SAM syndrome as well as residual MR, with no death or reoperation during follow-up, which may be beneficial to HOCM patients undergoing mitral subvalvular procedures with concomitant myectomy. In addition, no significant differences were observed between the two groups regarding baseline maximum LVOT gradients and the proportion of SAM syndrome. Comparatively, patients in the combined group had lower maximum LVOT gradients and a lower incidence of SAM at follow-up, suggesting that myectomy with concomitant mitral subvalvular procedures allowed the reduction of LVOT gradient and freedom from SAM more effectively in comparison with myectomy alone. NYHA functional class significantly decreased from the preoperative value and the clinical symptom improved in both groups, with no patients being class III or IV and no significant difference between groups, suggesting that both myectomy with concomitant mitral subvalvular procedures and myectomy alone achieved similar clinical benefits. Our results were consistent with that in previous literatures [9, 21, 22].

In this series including 50 HOCM patients undergoing mitral subvalvular procedures at the time of myectomy, no surgical death occurred. Unplanned aortic valve repair due to iatrogenic aortic valve perforation and unplanned repair of left ventricular rupture occurred in one of case each and were successfully treated. In principle, iatrogenic aortic valve perforation was prone to occur in young patients with small aortic roots. Left ventricular free wall rupture, a rare complication following myectomy, may be associated with excessive subaortic resection to the mitral anterior commissure. Although abnormal PMs were corrected, left ventricular free wall rupture was found via operative exploration to not be associated with the excision of muscle bundles. In this series, patients with left ventricular free wall rupture were successfully treated under cardiopulmonary bypass and cardioplegic arrest with a double-armed 3–0 polypropylene suture with a pledget placed in a horizontal mattress fashion, similar to the technique described to control a stab wound of the heart in close proximity myectomy to a coronary artery. Note that although HOCM patients with combined procedures had less septal hypertrophy, no iatrogenic septal perforation occurred, suggesting that less septal hypertrophy was not closely related to iatrogenic septal perforation during myectomy. Two patients with combined procedures developed complete atrioventricular block and required permanent pacemaker implantation. The incidence of pacemaker implantation (4.0%) was greater than expected based on other large series [14, 20]. This elevated incidence was attributed to the excessive subaortic resection to the side of the noncoronary valve. Notably, 44.0% of patients in the combined group developed the complete left bundle branch block following myectomy, which allowed the adoption of conservative surgical strategies in patients with preoperative right bundle branch block. In addition, no significant differences between two groups were found regarding immediate repeat surgery and major postoperative morbidity. No surgical death or iatrogenic septal perforation, together with no increase in major postoperative morbidity, may contribute to proving the safety of mitral subvalvular procedures during myectomy.

This study had some potential limitations. First, this was a single-center, observational study with a limited sample size, which may have influenced the generalizability of the results. Second, weighing the amount of the muscle/specimen following septal myectomy was performed in a minority of patients. Further study was warranted to evaluate the difference between the combined group and the alone group regarding the weight of excised muscle/specimen. Third, only a sizable minority of patients received cardiac magnetic resonance imaging, as it did not serve as a regular examination modality in the early times at our institution. Finally, mitral subvalvular anomalies may play an underestimated role in symptomatic HOCM patients, and further studies might determine its significance and identify the different genetic anomalies between HOCM patients with and without mitral subvalvular anomalies.

## 5. Conclusions

For HOCM patients without intrinsic MV diseases who are scheduled for surgery, a less pronounced septal hypertrophy may be closely associated with myectomy with concomitant mitral subvalvular procedures instead of myectomy alone. Mitral subvalvular procedures during myectomy are safe and allow the reduction of LVOT gradient and freedom from SAM more effectively in comparison with myectomy alone.

The echocardiographic image shows fibrotic and retracted secondary chorda inserted on the anterior mitral leaflet body (where the red arrow is pointing) and hypertrophic papillary muscle (where the blue arrow is pointing). LA, left atrium; AO, aorta; IVS, interventricular septum; and LV, left ventricle.

NYHA, New York Heart Association.

## Figures and Tables

**Table 1:** Baseline and surgical characteristics.

Variables	Combined group (*n* = 50)	Alone group (*n* = 131)	*p* value
*Demographics*			
Age (years)	52.9 ± 11.2	56.8 ± 11.8	0.045
Gender (male)	31 (62.0%)	66 (50.4%)	0.161
Recent smoking	17 (34.0%)	29 (22.1%)	0.101
*Concomitant diseases*			
Diabetes mellitus	5 (10.0%)	11 (8.4%)	0.772
Hypertension	20 (40.0)	51 (38.9%)	0.895
Cerebrovascular disease	3 (6.0%)	8 (6.1%)	>0.999
Family history of HCM	7 (14.0%)	8 (6.1%)	0.127
Family history of sudden death	4 (8.0%)	2 (1.5%)	0.049
*Preoperative cardiac status*			
NYHA functional class	—	—	0.034
II	11 (22.0%)	13 (10.0%)	—
III	36 (72.0%)	97 (74.0%)	—
IV	3 (6.0%)	21 (16.0%)	—
Atrial fibrillation	6 (12.0%)	12 (9.2%)	0.584
Right bundle branch block	1 (2.0%)	3 (2.3%)	>0.999
MV subvalvular anomalies	50 (100%)	0	—
False cords	13 (26.0%)	—	—
Retracted secondary chordae	34 (68.0%)	—	—
PM abnormalities	17 (34.0%)	—	—
*Echocardiographic data (TTE)*			
Maximum gradients (mmHg)	96.5 ± 17.8	92.2 ± 20.5	0.193
Septal thickness (mm)	16.4 ± 2.3	18.5 ± 3.2	<0.001
SAM	31 (100%)	180 (100%)	1.000
MR	3.0 (2.0–3.0)	3.0 (2.0–3.0)	0.472
LVEF (%)	67.5 ± 4.8	66.6 ± 4.2	0.217
LVEDD (mm)	44.7 ± 4.6	44.6 ± 4.4	0.893
*Procedures*			
Myectomy	50 (100%)	131 (100%)	1.000
ACC time (min)	38.1 ± 7.3	35.8 ± 7.5	0.065
Myectomy plus subvalvular procedures	50 (100%)	0	—
False cords cutting	13 (26.0%)	—	—
Retracted secondary chordae cutting	34 (68.0%)	—	—
PM release and/or resection	17 (34.0%)	—	—

Values are expressed as *n* (percent), mean ± standard deviation or median and interquartile range. HCM, hypertrophic cardiomyopathy; NYHA, New York Heart Association (classification); LVOT, left ventricular outflow tract; MV, mitral valve; PM, papillary muscle; TTE, transthoracic echocardiography; MR, mitral regurgitation; SAM, systolic anterior motion; LVEF, left ventricular ejection fraction; LVEDD, left ventricular endo-diastolic diameter; ACC, aortic cross-clamping.

**Table 2:** Independent association between baseline variables and concomitant subvalvular procedures.

Predictors	OR	95% CI	*p* value
Age	0.961	0.851–2.378	0.105
Males	1.607	0.826–3.127	0.184
Recent smoking	1.812	0.886–3.707	0.127
Family history of HCM	2.503	0.857–7.313	0.085
Family history of sudden death	4.109	0.994–9.651	0.058
NYHA class III and IV	2.460	0.961–5.677	0.084
Maximum gradients	1.053	0.912–3.124	0.176
Septal thickness	0.887	0.612–0.917	0.005

OR, odds ratio; CI, confidence interval.

**Table 3:** Perioperative outcomes.

Variables	Combined group (*n* = 50)	Alone group (*n* = 131)	*p*
*Intraoperative*			
Immediate repeat surgery	3 (6.0%)	7 (5.3%)	>0.999
Inadequate septal myectomy	1	4	—
Left ventricular free wall rupture	1	2	—
Aortic valve perforation	1	0	—
Septal perforation	0	1	—
TEE data			
Maximum gradients (mmHg)	8.1 ± 5.2	11.9 ± 7.0	<0.001
Septal thickness (mm)	13.7 ± 1.7	14.3 ± 2.6	0.132
SAM	0	17 (13.0%)	0.007
MR	1.0 (0-1.0)	1.0 (1.0–1.0)	0.086

*Postoperative*			
Surgical death	0	1 (0.8%)	0.536
Complete atrioventricular block	2 (4.0%)	4 (3.1%)	0.669
Previous right bundle branch block	1	2	
New-onset atrial fibrillation	2 (4.0%)	3 (1.7%)	0.617
Complete left bundle branch block	22 (44.0%)	53 (40.5%)	0.665
Cerebrovascular adverse events	1 (2.0%)	2 (1.5%)	>0.999
Prolonged ventilation (>72h)	1 (2.0%)	3 (1.7%)	>0.999
Postoperative hospital stay (d)	6 (5-6)	6 (5-6)	0.412

Values are expressed as *n* (percent), mean ± standard deviation or median and interquartile range. TEE, transesophageal echocardiography.

## Data Availability

The data used to support the findings of this study are available from the corresponding author upon request.
